# Cardiovascular Risk Factors from Early Life Predict Future Adult Cardiac Structural and Functional Abnormalities: A Systematic Review of the Published Literature

**DOI:** 10.12970/2311-052X.2014.02.02.4

**Published:** 2014-07

**Authors:** Arjun K. Ghosh, Darrel P. Francis, Nishi Chaturvedi, Diana Kuh, Jamil Mayet, Alun D. Hughes, Rebecca J. Hardy

**Affiliations:** 1 International Centre for Circulatory Health, Imperial College London; 2 Medical Research Council Unit for Lifelong Health and Ageing, London, UK

**Keywords:** Life course Cardiology, echocardiography, blood pressure, glucose, body mass index

## Abstract

**Background:**

Clinical practice evaluates cardiovascular risk based on current risk factor (RF) levels [Blood pressure (BP), body mass index (BMI) and glycaemic control] largely disregarding previous risk-factor history over the totality of the life course. RFs are related to contemporaneous echocardiographic measures of cardiac structure and function which in turn are independently related to cardiovascular morbidity and mortality in cross-sectional studies. However, the effect of lifetime or earlier RF history on future echocardiographic changes has never been systematically examined.

**Methods:**

A systematic review of the published literature identified 24 studies relating either earlier BP, BMI, glycaemic control or a combination to future cardiac structure and/or function.

**Results:**

The majority of studies showed that elevated BP and BMI in earlier life and greater cumulative burden of these factors resulted in worse cardiac structure up to 24 years later. Studies examining glycaemic control as RF were few, but poorer glycaemic control in young adults was associated with increased future left ventricular mass.

While only 5 papers related RFs to future cardiac function, all RFs were positively associated with worse future diastolic function.

**Conclusions:**

BP, BMI and glycaemic control measures in childhood, adolescence and early adulthood and subsequent longitudinal trajectories of BP and BMI are predictive of future abnormalities in cardiac structure and function. Lifetime RF history should be used to inform clinical practice. Further research is required to enable the identification of any sensitive periods in the life course to enable prevention when it is most likely to be effective.

## Introduction

Clinical practice is to evaluate cardiovascular risk based on current risk factor levels largely disregarding previous risk-factor history over the *totality* of the life course. This is because there is a large clear literature associating current risk with cardiovascular morbidity and mortality [1]. There is also literature cross-sectionally associating current risk factors with echocardiographic changes and outcomes with these changes relating to cardiovascular events [2]. However, the effect of *lifetime* risk factor history on *future* echocardiographic changes has never been systematically examined.

Several cardiovascular risk factors correlate with echocardiographic changes and outcomes in cross-sectional studies. Elevated blood pressure (BP) and body mass index (BMI) have been associated cross-sectionally with increased left ventricular mass (LVM) and, in some cases, with left ventricular hypertrophy (LVH) [3] and both elevated LVM and LVH are independent predictors of cardiovascular morbidity and mortality [2, 4]. There has also been some research relating presence or absence of diabetes to later echocardiographic outcomes [5]. However, less is known about how earlier-life levels of these risk factors, changes in risk factors, and duration of and long-term control of hypertension, obesity and diabetes is associated with subsequent cardiac morphology and function [6].

A life course approach to epidemiology investigates long-term biological, behavioural and psychosocial processes that link adult health and disease risk to physical and social exposures occurring in gestation, childhood, adolescence, earlier in adulthood or across generations [7]. Such an approach acknowledges the importance of the established adult Coronary Heart Disease (CHD) risk factors to later health, but adds a longitudinal aspect by considering the continuity and tracking of these risk factors across the life course [8, 9].

There is a growing appreciation that exposures to established CHD risk factors (e.g. elevated BP, BMI and poor glycaemic control) from conception onwards through childhood and early adulthood can influence later cardiovascular morbidity and mortality and therefore, potentially cardiac structure and function [10, 11]. However what remains unclear is whether detrimental changes in cardiac structure and function are caused by exposures at particularly sensitive points in an individual's life, the overall cumulative burden of exposure, or accelerated change during particular phases of life. For example, it is unknown whether being overweight from the age of 20 years onwards is more detrimental to the heart at the age of 60 years, than first becoming overweight at the age of 50 years, or whether for individuals with the same current BP, the effect on cardiac structure and function varies depending on whether there had been a gradual increase in BP over a 30 year period or a steeper increase over a shorter period.

We conducted a systematic review of the published literature to determine if increasing lifetime exposure to raised BP, BMI and impaired glycaemic control was associated with worse future cardiac structure and function.

## Methods

### Criteria for Study Inclusion and Search Strategy

Studies eligible for inclusion were observational studies with *longitudinal repeated* measures of one or more of the exposures of interest (BP, BMI and glycaemic control) and *at least one* echocardiographic examination of participants (looking at cardiac structure and/or function) in adulthood. Eligible study samples consisted of individuals who were healthy, non-disabled and community-dwelling at the time of measurement of the risk factors, the risk factors having being analysed as continuous variables.

Outcomes considered were any echocardiographic measures of cardiac structure (e.g. wall and chamber dimensions and volumes) or function (e.g. fractional shortening (FS), E/A ratio, and tissue Doppler based measures such as E/e’ ratio).

**Figure 1:**
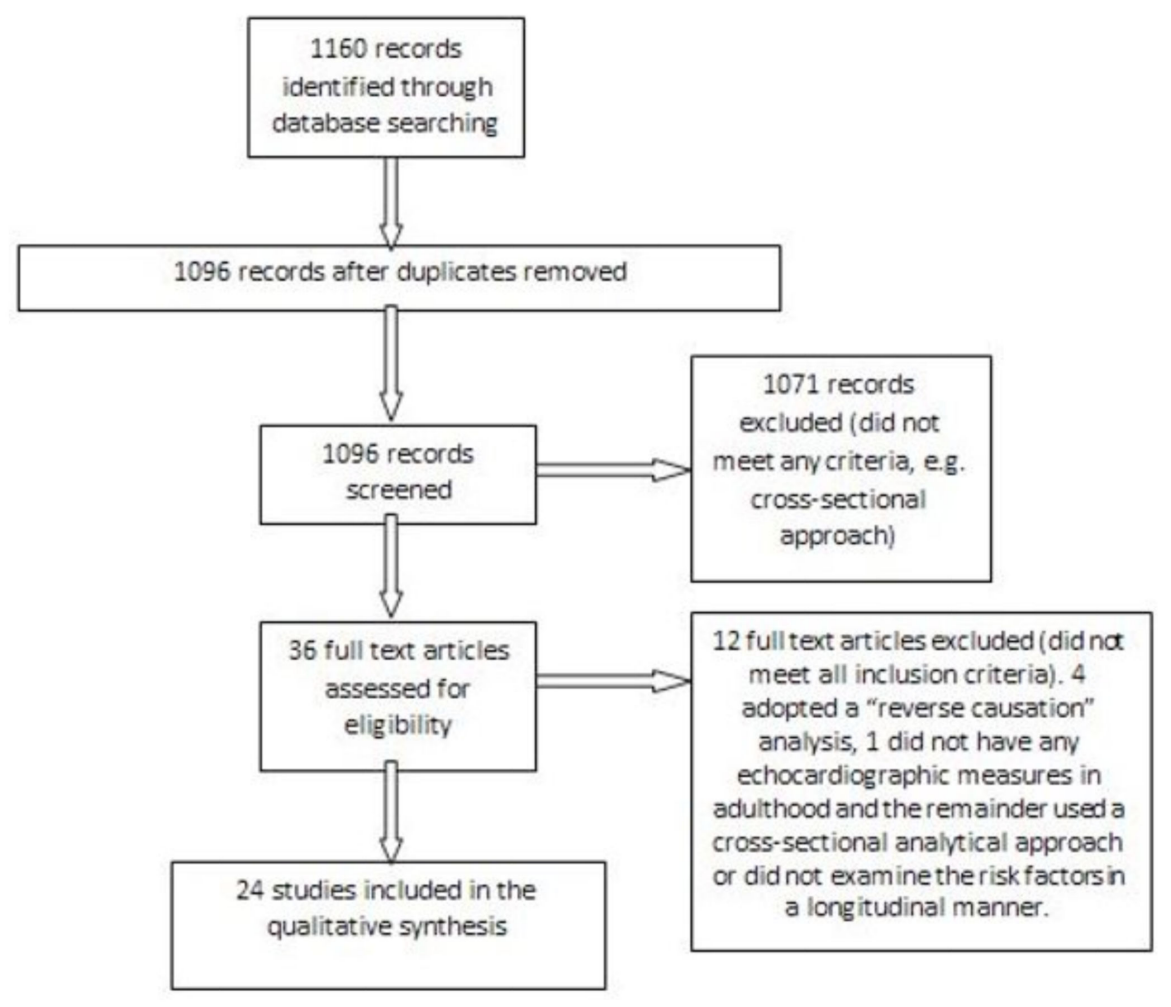
Flow of studies through the review (after the PRISMA and MOOSE statements) [12, 13].

Searches of the electronic databases PubMed and EMBASE (January 1966 to October 2013) were performed using the following Mesh terms: (longitudinal OR cohort OR life course OR lifecourse OR follow up OR tracking) AND (blood pressure measurements OR body mass index measurements OR birth weight OR lipids OR glycaemic control) AND (echocardiography). The detailed search strategy is outlined in the online supplemental material. Figure 1 summarises the identification of studies. There were 1096 unique records which were screened. A total of 24 papers were identified using a screening questionnaire after screening the full text of 36 potentially eligible papers. Any uncertainty about study eligibility was resolved through discussion between 3 of the authors. Details of relevant published results, study population, measurement of risk factors and outcome and details of statistical analysis were extracted from the papers using a data extraction form.

The analyses presented in the papers were too varied to be able to carry out a formal meta-analysis of the published data. Furthermore, the cardiac outcomes assessed and the ages at which risk factors were measured were too heterogeneous to carry out an individual level meta-analysis. Hence, no attempts were made to contact authors for additional information.

## Results

The 24 papers meeting the inclusion criteria used data from 13 different cohort studies. The characteristics of included studies are provided in Table 1. The full main findings and statistical methods used are provided in Table S1 in the online supplemental material. There was more than 1 paper from 5 studies; Framingham (2 papers), Framingham Offspring (4), Bogalusa (4), Uppsala (4) and CARDIA (2). These papers focussed on different risk factors and/or outcomes and were thus all included in the review. The range of follow up periods in these studies was wide, varying from 2 years to 70 years and the mean age of study members at baseline varied from 7 to 89. Most studies were conducted in adult participants, although 4 measured risk factors in children with follow up into adulthood (Bogalusa, Hertfordshire cohort and the studies by Johnson and Kumaran) [14–17]. Six studies were in men only (Uppsala cohort studies and in the studies by Ridderstrale, Vijayakumar, Jokiniitty, Strand and Zureik) [17–25], but the majority had male and female participants. Bogalusa and CARDIA had a purposely bi-ethnic study population, while Kumaran's study was on South Asian Indians and Lin's study on Chinese seniors. The others consisted of European-origin population cohorts.

The longitudinal relationship between risk factors and future cardiac structure and function was investigated in a variety of ways. Some related risk factors at a single point in time with future cardiac structure [3, 15, 16, 18, 20, 22–24, 26–29] we have called this the “prospective approach” for the purposes of this review. Others, with regularly repeated measures of risk factors, calculated summary measures of the risk factors over a period of time for each individual, for example the area under the curve (AUC) or rate of change, and related those to future cardiac structure and function [21, 29–31]. We have grouped these studies into an “accumulation or change approach” category.

A third approach used in the Framingham Offspring papers concentrated on investigating risk factors in relation to longitudinal changes in cardiac measures, rather than relating changes in risk factors to cardiac measures at a single time point [32–35].

### Cardiac Structure

#### Blood Pressure

The relationship between BP and cardiac structure was investigated in all 13 cohorts.

##### Prospective Approach

BP measured at different times across the lifetime has been positively related to future left ventricular (LV) wall thickness and mass.

Childhood Systolic and Diastolic BP (SBP and DBP) (average age 13.3 years) were positively associated with future LVM (4 years later) and LVH (19 years later) in Bogalusa but SBP was not associated with LV dilation 19 years later [16, 27, 29]. Similar positive associations between SBP and mean arterial pressure (MAP) and future LVM (measured 10-20 years later) were also seen in young adults (18-30 years in age) [23, 26].

Three studies considered the association between SBP at older ages and subsequent cardiac structure. In one study SBP and DBP at age 50 years were associated with an increased prevalence of LVH at age 70 years [24] while in another DBP at age 42.1 years positively predicted LVM 20 years later [18]. Finally, in a study of elderly Chinese, only baseline pulse pressure (PP) was positively associated with LVH 4 years later [28].

##### Accumulation or Change Approach

Studies have shown a positive relationship between SBP change across the life course and future cardiac structure.

2 studies investigated the effect of rate of change in SBP in children or young adolescents. Greater LVM at age 33 years was associated with a greater cumulative SBP burden over a prior 23 year period (quantified by AUC) [30], while greater wall thickness [(interventricular septal diameter (IVSD) and left ventricular posterior wall thickness (LVPWT)] but not left ventricular internal diameter (LVID) in 21 to 24 year olds was associated with sustained higher BP over the preceding 5 years [14].

In young black men and black women a greater increase in SBP over 10 years was positively associated with future LVM index (LVMI) [31].

Similar relationships between sustained hypertension or increased SBP and future wall thickness were also seen in older individuals - in Uppsala (50 y at baseline, 20 y follow up) [21] and in a Parisian cohort (47-58y at baseline, 2y follow up) [25].

#### BMI and Weight

##### Prospective Approach

Greater BMI has been associated with greater future LVM, however this relationship is more complex when very early BMI or weight is considered.

The relationship between weight at birth or in infancy with later cardiac structure has been investigated in 2 studies.

Birth weight was not associated with LVMI or left ventricular geometry (LVG) at a mean age of 49.5 years, and a longer length at birth showed a weak and non-significant association with increasing LVMI, LVG and relative wall thickness (RWT) [15]. However in the study with the longest follow-up, greater weight at 1 year was associated with lower LVM, IVSD, LVPWT and RWT, but was not associated with LVID at a mean age of 70 years [17].

When baseline BMI or weight in children and young adolescents was positively related to future cardiac structure, it was found to be associated with future LVM [29], LV dilation, eccentric LVH and left atrial diameter (4 years later) [16, 27]. BMI in young adults was also positively associated with LVM 10 or 20 years later [23, 26.

BMI was also related to future cardiac structure in 2 older cohorts where it was predictive of future LVMI after 4 or 10 years (baseline ages 35-45 and 71.7 years) [22, 28]. High BMI at the age of 50 years was also associated with increased prevalence of LVH and higher left atrial diameter at 70 years [20, 24].

##### Risk Factor Accumulation or Change Approach

Relating increases in BMI to future LV structure has only been carried out in young and older adults. A greater cumulative BMI burden (AUC) over a period of 23 years was associated with a higher LVM at 33 years [30]. Among black men and black and white women, greater increases in BMI over a 10 year period, were associated with a greater LVMI at ages 28-40 years [26].

In older individuals (52y old men) BMI changes were not associated with changes in LV structure. However follow up here was only for 2 years [25].

### Glycaemic Control

Although no relationships were found between childhood glycaemic control and later cardiac structure, this was not the case for later measures of glycaemia.

Only 4 studies have looked at the prospective effect of glycaemic control on cardiac structure [16, 20, 23, 28].

In children, fasting glucose was not associated with LVH 24 years later [16]. However in 20 year olds, baseline insulin was associated with LVMI measured 20 years subsequently.

Relationships between glycaemic control and cardiac structure were evident in older cohorts. Proinsulin levels at age 50 years were positively correlated with left atrial diameter at age 70 years [20], while fasting glucose at baseline was positively correlated with year 4 LVMI in Chinese seniors [28].

### Longitudinal Modelling of Cardiac Outcomes

Instead of modelling changes in risk factors, the Framingham Offspring Study papers have concentrated on relating risk factors to longitudinal changes in cardiac measures over 16 year and 4 year periods [32–35].

Higher SBP was associated with higher LVMI, and higher SBP and PP were significantly related to greater LV dimensions and wall thickness as well as larger left atrial diameter [34]. The relationships between BP and change in aortic root diameter were more complex with lower SBP and PP and higher DBP and MAP being associated with higher follow-up aortic root diameter [35].

BMI was also related to changes in cardiac structure with positive associations with LVID, LV wall thickness [32] and LVM, the last association being stronger in women [34]. Higher BMI was also positively associated with greater aortic root diameter [33].

### Cardiac Function

Although the number of studies investigating the effect of risk factors on future cardiac function was limited, BP, BMI and glycaemic control were all predictors of future cardiac function.

A total of 9 studies investigated cardiac function of which 4 were excluded from this section as analyses were carried out in a cross-sectional manner or only on a subgroup of the study cohort [18, 23, 27, 28]. Of the 5 papers investigating longitudinal associations, 3 were on the Uppsala cohort [14, 19–21, 32].

Sustained higher SBP over a 5 year period in adolescents and young adults was positively associated with fractional shortening (FS) and mean velocity of circumferential fibre shortening, measured at ages 21-24 years [14]. In the Framingham Offspring cohort, SBP was positively associated with changes in FS [32].

In older individuals, although BP was not shown to have a relationship with ejection fraction (EF), higher SBP and DBP at age 50 years were associated with decreased E/A ratio and higher SBP to higher A-wave [20, 21].

BMI and glycaemic control were only considered in the Uppsala cohort. Higher BMI at age 50 was associated with diastolic dysfunction manifest as decreased E/A ratio and larger A-wave after adjustment at age 70 [20]. Fasting glucose at 50 years was negatively related to EF at age 70, and positively correlated with E-wave [19], and specific insulin and proinsulin levels were positively correlated with the magnitude of the A-wave [20].

## Discussion

Most studies included in the review show associations between higher SBP and BMI at an earlier time-point and poorer cardiac structure measured up to 70 years later. Single measures of SBP and BMI measured in childhood, adolescence and early adulthood and greater cumulative burden of these risk factors predict cardiac measures (LVM, LV wall thickness) in adulthood in the few studies that look at this. There is a lack of studies relating birth weight, infant weight or glycaemic control to cardiac structure and a lack of studies considering the longitudinal relationship of any risk factor with cardiac function.

### Assessment of Bias and Justification for Exclusions

Due to the variation in cardiac measures investigated, the variation in age at measurement of risk factors and length of follow-up, and the differences in statistical analysis with different levels of adjustment for potential confounding variables, it was not possible to perform a meta-analysis of results. We were thus unable to formally assess publication bias. By systematically reviewing the literature according to published guidelines and following a pre-specified protocol, we believe we have included all published results of the main associations of interest. However, we did limit our search to two electronic databases and to studies written in English and therefore may have missed some relevant studies.

### Interpretation of the Findings

Ideally, life course studies require risk factors to be collected over a substantial period of time and at various phases of the life course. The few studies that have shown single risk factor measurements in childhood, adolescence or early adulthood to be correlated with adult echocardiographic measures may reflect “tracking” of the risk factor from childhood to adulthood [36]. Few studies investigate this possibility and so it remains unclear whether these observations can be interpreted as showing a distinct contribution from earlier exposure to risk factors. Current SBP seemed to account for the effect of SBP measured 8 years earlier in the only study that investigated whether prior SBP had a predictive value independent of current SBP [37]. Due to the availability of only a single related study, whether SBP measured earlier in the life course, especially in childhood, adolescence or early adulthood, may demonstrate an independent role (by affecting change in a particularly sensitive period) remains unclear.

Other studies suggest a “cumulative” effect of exposure to obesity and hypertension on later echocardiographic results (possibly through an accrual of damage over time worsened by prolonged exposure). However, no study has analysed the impact of risk factor trajectories starting from birth and continuing on into early, mid and later adulthood on cardiac structure and function at an older age. Neither of the two studies investigating very early weight and adult LVM found evidence of an association, suggesting that fetal development associated with low birth weight in the normal range exerts minimal effect on future LVH. However, both studies were quite small and may have lacked the required statistical power to detect a relatively small relationship.

While this review focuses on the longitudinal effect of BP, BMI and glycaemic control on cardiac structure and function, the possibility of reverse causality cannot be ruled out. Some studies have explored the effects of an echocardiographic diagnosis (e.g. LVH) on subsequent levels of BP. These studies have shown that increased LVM, in normotensive individuals or subjects with optimally controlled blood pressure, predicts the development of future hypertension [38, 39]. Poorer cardiac function in the form of higher EF has also been found to be associated with higher subsequent SBP [40].

Some studies suggest that 24 hour PP, as opposed to SBP, is more predictive of later LVM [22]. Different BP parameters (SBP, DBP, PP, MAP) reflect the effect of BP in subtly different ways. For example, PP depends on stroke volume and arterial stiffness whereas MAP is determined by cardiac output and peripheral resistance [41, 42]. From the papers reviewed, it is not possible to determine which measures are most useful as predictors over the longer term, as different measures have not been compared against each other. Consideration of these multiple BP measurements in future longitudinal studies may result in better insights into the hemodynamic mechanisms acting over the life course to affect cardiac structure and function.

The studies in our review showed associations between poorer glycaemic control and worse left ventricular mass, ejection fraction and E/A ratio [16, 20, 23, 28]. However caution would be needed before extrapolating these findings to all population groups due to the small number of studies. In addition, the effect of *long-term* glycaemic control was not measured and although attempts were made to account for the role of confounding factors, it is difficult to tease out the effect of glycaemia independent of its effect through BMI.

### Implications of the Findings

One of our striking findings was the lack of a standardised approach to analysis. The papers in this review were published between 1983 and 2010, and the analytical approaches have developed in line with the changing nature of the hypotheses with regard to biological mechanisms. Multivariable linear regression, relating the risk factor at a single time point to a subsequent echocardiographic measure, has been used in most studies. However, analyses become more complex if the aim is to use repeated measures of the risk factor. A few studies have attempted to deal with problems of modelling correlated measures of exposure by creating a summary measure for each individual e.g. AUC, or rate of change [43], and relating these to the outcome [44]. However, no studies have as yet attempted to disentangle the effect of “cumulative” risk factor burden from exposures during sensitive periods [45, 46]. Ultimately, there is no standard statistical approach to analysis linking a repeated exposure to a subsequent health outcome, and the most appropriate method will depend on the specific hypothesis under study and hence the plurality of techniques used in the included papers [47].

Another issue is the added complexity when children are followed up into early adulthood as childhood growth may not be adequately captured by changes in childhood BMI [48]. Fat mass and lean mass may need to be considered separately when analysing cardiac growth in children, but most studies only have measures of BMI and are thus not able to consider this [49, 50].

We also found a lack of studies dealing with the longitudinal effects of risk factors on cardiac function, and the few that have, investigate different aspects of systolic or diastolic function. Many of the populations included in the review have been young adults and as such, the vast majority of study subjects have had normal cardiac function. As a result, it is not possible to make a generalised systematic assessment of the findings and this area would benefit from further research.

Some findings have also been different among blacks and whites in the bi-ethnic studies. Further research with more heterogeneous study populations would be helpful in making findings more generalizable.

New research on the long-term effect of exposure to poorly controlled BP, BMI and glycaemia is warranted to better understand the mechanisms by which they independently, cumulatively or interactively affect later cardiac function and structure. The life course approach could help identify periods where exposures to risk factors may be particularly detrimental to cardiac function and structure. This new knowledge would be invaluable to inform policy related to cardiovascular disease prevention, enabling earlier targeted intervention to individuals at risk.

## Conclusions

This systematic review provides some evidence that earlier elevated BP, high BMI and poor glycaemic control result in worse cardiac structure and function up to 24 years later. This information should be used to inform clinical practice, but it should be remembered the number of studies on which this evidence is based is limited, and the findings are difficult to generalize to all population groups. There is a lack of studies investigating either the effect of changes in risk factors over the life course or the cumulative effect of exposure to risk factors on cardiac structure and function. Future studies utilizing a life course approach, to identify if there are sensitive periods where exposure is particularly detrimental, might suggest time-points when prevention may be best targeted. Ultimately such information would be invaluable from a public health and preventive Cardiology perspective.

## Supplementary Material

The supplemental materials can be downloaded from the journal website along with the article.

Supplementary data

## Figures and Tables

**Table 1:** Characteristics of Studies Included in the Systematic Review

Paper: First author and year	Study name and location	Analysed sample size (at last examination round if not further qualified)	Mean Age +/- 1SD(Age range)in years at last follow up (unless otherwise mentioned)	Maximum duration of follow up in years and maximum number of repeated examination rounds for the purpose of quoted paper	Repeat measure(s) related to echocardiographic outcome for the purpose of quoted paper (not all measures may have been taken in all rounds)	Cardiac Outcome measure(s)
Johnson 1983	Bourbon County, USA	837 at baseline; 77 men and 51 women qualified for final inclusion	16-19 at baseline	5 and 1	BP*, BMI†	Structure and function
Lauer 1991	Framingham, USA	451 (152 men and 299 women)	68 +/- 6	30 and 16	BP, BMI	Structure
Urbina 1995	Bogalusa, USA	90 males and 70 females at first echocardiography round	13.3 at first echocardiography round (9-22)	4 with 1 follow-up round of echocardiography	BP, weight, height	Structure
Vasan 1995	Framingham, USA	2803 men and 3411 women at baseline; 1849 men and 2152 women at 1^st^ follow up	20-89 at baseline	8 and 1	BP, BMI	Structure
Vijayakumar 1995	Hertfordshire Cohort, UK	290 men	66.9 +/- 3.2	70 and 2	BP, BMI, glucose and insulin	Structure
Zureik 1995	Paris, France	177 men	51.6 +/- 2.9 (47-58)	2 and 1	BP, BMI	Structure
Kumaran 2000	Mysore, India	435 (237 men and 198 women)	49.5 +/- 4.8	49.5 and 1	BP, BMI	Structure
Arnlov 2001	Uppsala, Sweden	2330 at baseline, 1227 at 1^st^ follow-up (431 men qualified for inclusion)	48-51 in the 1^st^ round	20 and 1	Glycaemic control	Function
Jokiniitty 2001	Tampere, Finland	97 men at baseline and 86 men at follow up	35-45	10 and 1	BP, BMI	Structure
Sundstrom 2001	Uppsala, Sweden	2322 men at baseline; 1221 at follow-up (475 qualified for inclusion)	70-74	20 and 1	BP, BMI, glycaemic control	Structure
Bjorklund 2002	Uppsala, Sweden	2322 men at baseline; 1221 at follow-up (583 qualified for inclusion)	70	20 and 1	BP	Structure and function
Gardin 2002	CARDIA, USA	5115 at baseline; 4243 at 1^st^ follow-up; 1536 at 2^nd^ follow-up; 1189 qualified for inclusion (544 men and 645 women)	18-30 at baseline	10 and 2	BP, BMI	Structure
Lorber 2003	CARDIA, USA	737 men and 881 women	18-30 at baseline	10 and 5	BP, BMI, fasting insulin levels	Structure
Li 2004	Bogalusa, USA	1420 at baseline;467 at 6^th^ follow-up(182 men and 285 women)	32.6 (20-38)	23 and 2 to 12 follow-ups with anaverage of 6	BP, BMI	Structure
Arnlov 2005	Uppsala, Sweden	2330 at baseline; 1227 at follow-up (505 men qualified for inclusion)	70	20 and 1	BP, BMI, glycaemic control	Structure and function
Haji 2006	Bogalusa, USA	197 men and 309 women	32 +/- 3	23 and 2 to 12 follow-ups with an average of 6	BP, BMI, fasting glucose	Structure and function
Strand 2006	Oslo, Norway	56 men	42.1 +/- 0.5 at baseline	20 and 1	BP, BMI	Structure and function
Lin 2007	The Longitudinal Study of Aging, China	193 at baseline; 170 at 1^st^ follow-up; 144 at 2nd follow-up (81 men and 24 women qualified for inclusion)	71.7 +/- 3.9 (60-81)	4 and 2	BP, BMI, glycaemic control	Structure and function
Toprak 2008	Bogalusa, USA	338 men and 486 women	36 (24-44)	24 and 1	BP, BMI, glucose	Structure
Lieb 2009	Framingham Offspring, USA	1973 men and 2244 women in long term study; 1094 men and 1511 women in short term study	45	4 to 16 and maximum of 4 follow-ups	BP, BMI, diabetic status	Structure
Cheng 2010	Framingham Offspring, USA	1851 men and 2211 women	45 +/- 10 at baseline	16 and 4	BP, BMI, fasting glucose	Structure and function
Lam 2010	Framingham Offspring, USA	1671 men and 1835 women in short term group; 2187 men and 2355 women in long term group	52+/-10 for men and 51+/-10 for women in short term group; 46+/-10 for men and 45+/-10 for women in long term group (25-74)	4 to 16 and 2-6 follow-ups	BP, BMI	Structure
McManus 2010	Framingham Offspring, USA	2102 men and 2301 women in long term group; 1559 men and 1806 women in short term group	45	4 to 16 and maximum of 4 follow-ups	BP, BMI, diabetic status	Structure
Ridderstrale 2010	Military recruits, Sweden	74 male army recruits at baseline, 64 at follow-up	40	20 and 1	BP, BMI,	Structure and function

* Blood Pressure

† Body Mass Index.
