# Supplementary material for: Cardiovascular Risk Factors from Early Life Predict Future Adult Cardiac Structural and Functional Abnormalities: A Systematic Review of the Published Literature
Source: J Cardiol Ther. Author manuscript; Available in PMC 2016 Jun 10. (PMC4902124; doi:10.12970/2311-052X.2014.02.02.4)
Supplement: Supplementary data [file NIHMS61616-supplement-Supplementary_data.docx]

**Supplementary material**

**Supplementary methods**

The electronic databases PubMed and EMBASE were searched to identify potentially eligible published studies, abstracts, review articles and letters. The following Mesh terms were used in the searches (longitudinal OR cohort OR life course OR lifecourse OR follow up OR tracking) AND (blood pressure measurements OR body mass index measurements OR birth weight OR lipids OR glycaemic control) AND (echocardiography). The searches included studies from January 1966 to October 2013 and the results were limited to articles published in English. After removing duplicates, 1096 study abstracts were screened resulting in the exclusion of 1071 papers that did not meet inclusion criteria (were cross-sectional studies, hospital-based, measured outcomes other than echocardiographic parameters, or the risk factors were not analysed as continuous variables). The full text of 36 articles were obtained and reviewed for eligibility using a standardised inclusion/exclusion form, and 12 were excluded as being ineligible. Of the 12 that were excluded, 4 had adopted a “reverse causation” analysis,^1-4^ 1 did not have any echocardiographic measures in adulthood^5^ and the remainder used a cross-sectional analytical approach or did not examine the risk factors in a longitudinal manner.^6-12^

Data extraction forms were used to extract data from the remaining 24 articles.

It was considered unlikely that a meta-analysis would be possible because of the anticipated differences in statistical approach, measurement of risk factors and cardiac outcomes.

Reference List

(1) de Simone G, Devereux RB, Chinali M, Roman MJ, Welty TK, Lee ET, Howard BV. Left ventricular mass and incident hypertension in individuals with initial optimal blood pressure: the Strong Heart Study. *J Hypertens* 2008;26(9):1868-74.

(2) Grossman C, Grossman A, Koren-Morag N, Azaria B, Goldstein L, Grossman E. Interventricular septum thickness predicts future systolic hypertension in young healthy pilots. *Hypertens Res* 2008;31(1):15-20.

(3) Ingelsson E, Pencina MJ, Levy D, Aragam J, Mitchell GF, Benjamin EJ, Vasan RS. Aortic root diameter and longitudinal blood pressure tracking. *Hypertension* 2008;52(3):473-7.

(4) Post WS, Larson MG, Levy D. Impact of left ventricular structure on the incidence of hypertension. The Framingham Heart Study. *Circulation* 1994;90(1):179-85.

(5) Zureik M, Bonithon-Kopp C, Lecomte E, Siest G, Ducimetiere P. Weights at birth and in early infancy, systolic pressure, and left ventricular structure in subjects aged 8 to 24 years. *Hypertension* 1996;27(3 Pt 1):339-45.

(6) Burke GL, Arcilla RA, Culpepper WS, Webber LS, Chiang YK, Berenson GS. Blood pressure and echocardiographic measures in children: the Bogalusa Heart Study. *Circulation* 1987;75(1):106-14.

(7) Devereux RB, Roman MJ, Liu JE, Lee ET, Wang W, Fabsitz RR, Welty TK, Howard BV. An appraisal of echocardiography as an epidemiological tool. The Strong Heart Study. *Ann Epidemiol* 2003;13(4):238-44.

(8) Gardin JM, Wagenknecht LE, Anton-Culver H, Flack J, Gidding S, Kurosaki T, Wong ND, Manolio TA. Relationship of cardiovascular risk factors to echocardiographic left ventricular mass in healthy young black and white adult men and women. The CARDIA study. Coronary Artery Risk Development in Young Adults. *Circulation* 1995;92(3):380-7.

(9) Kupari M, Koskinen P, Virolainen J. Correlates of left ventricular mass in a population sample aged 36 to 37 years. Focus on lifestyle and salt intake. *Circulation* 1994;89(3):1041-50.

(10) Soto LF, Kikuchi DA, Arcilla RA, Savage DD, Berenson GS. Echocardiographic functions and blood pressure levels in children and young adults from a biracial population: the Bogalusa Heart Study. *Am J Med Sci* 1989;297(5):271-9.

(11) Strand A, Kjeldsen SE, Gudmundsdottir H, Os I, Smith G, Bjornerheim R. Tissue Doppler imaging describes diastolic function in men prone to develop hypertension over twenty years. *Eur J Echocardiogr* 2008;9(1):34-9.

(12) Toprak A, Wang H, Chen W, Paul T, Ruan L, Srinivasan S, Berenson G. Prehypertension and black-white contrasts in cardiovascular risk in young adults: Bogalusa Heart Study. *J Hypertens* 2009;27(2):243-50.

**Supplementary Table**

**Table 2. Main findings and statistical methods used**

| **Paper: First author and year** | | **Study name and location** | **Main findings** | **Statistical methods used** |
| --- | --- | --- | --- | --- |
| Vijayakumar  1995 | | Hertfordshire  Cohort, UK | Weight: Higher weight at 1 year associated with lower LVM* at a mean age of 66.9 years, after adjustment for confounding factors. Higher weight at 1 year positively associated with IVSD^†^, LVPWD^‡^ and RWT^§^ but not with LVIDD^\|\|^, LVIDS^#^ and cardiac output before and after adjustment for BSA**. Higher birth weight non-significantly associated with decreasing LVM. | Multiple regression analysis relating weight at 1 year to LVMI^††^ adjusted for adult BSA, SBP^‡‡^ and age. |
|  | |  |  |  |
| Kumaran  2000 | | Mysore, India | Weight: Birth weight (when adjusted for sex) was not associated with LVMI or with LVH^§§^ at a mean age of 49.5 years. Longer length at birth length was associated with increasing LVMI, LVH and RWT. | Linear regression and logistic regression relating birth size measures to LVM and LVH, respectively. |
|  | |  |  |  |
| Johnson  1983 | | Bourbon County, USA | BP ^\|\|\|\|^: Higher sustained 5 year SBP between 16-19 years and 21-24 years (follow-up measures used but due to high tracking and selection carried out this can be interpreted as sustained high SBP) associated with higher FS and mean velocity of circumferential fibre shortening. IVSD, LVPWTD^##^ and LVPWTS*** significantly less in those with sustained low SBP versus those in intermediate and higher SBP groups. LVIDD and LVIDS did not differ among groups.  BMI ^‡‡‡^: Measures of body size used as correcting factors in the analyses. | BP measured at baseline (16 to 19 years) and selected sample (those above 95^th^, below 5^th^ percentile of SBP at baseline and a random sample of the rest) measured again 5 years later. High, intermediate and low SBP groups defined in terms of follow-up SBP and ANOVA ^†††^ used to compare echocardiography measures across the three groups. |
|  | |  |  |  |
| Urbina  1995 | | Bogalusa, USA | BP: Higher SBP and DBP at the time of first examination (average age of 13.3 years, age range 9 to 22 years) predicted higher LVM at follow-up 4 years later.  Weight: Higher adiposity (weight, ponderal index, triceps skin fold thickness) at first examination predicted higher LVM at follow up in univariable analyses. In multivariable analyses only baseline weight was significant in determining final LVM consistently across both sexes. | ANOVA used to look at sex and race differences and interactions. Tracking coefficients for anthropometric measures, BP and LVM by sex derived and colinearity tested for. |
| Li  2004 | | Bogalusa, USA | BP: Higher SBP in childhood and greater cumulative burden from childhood to adulthood (AUC^§§§^ over a 23 year period) associated with higher LVM at an average age of 32.6 years (age range 20-38 years).  BMI: Higher BMI in childhood and greater cumulative burden from childhood to adulthood (AUC) associated with higher LVM at 23 year follow-up. | Multiple regression with LVM as outcome and standardized risk factor measures since childhood and AUC of risk factors as explanatory variables. |
| Haji  2006 | | Bogalusa, USA | BP: Childhood BP (mean age 11 +/-3 years) not associated with adult LV^\|\|\|\|\|\|^ dilation (mean age 32 +/-3 years).  BMI: BMI in childhood positively predicted LV dilation in adulthood.  Cardiac function analysed in cross-sectional manner. | RFs ^###^ measured at first and last examinations used as the childhood and adulthood values. Multiple logistic regression used with RFs corrected for age, gender and race. |
| Toprak  2008 | | Bogalusa, USA | BP: DBP**** levels in childhood (5 to 18 years, average age 12 years) significant positive predictor of concentric LVH in adulthood (24 to 44 years, average age 36 years, 24 years after childhood examinations)  BMI: Higher BMI in adulthood and childhood significant determinants of developing eccentric LVH.  Glycaemic control: Childhood diabetes mellitus was not associated with adult cardiac structure. | ANCOVA was used to evaluate differences between 4 LVG^††††^ groups. Adjusted multinomial logistic regression analyses performed to define adult and childhood determinants of LVG in adulthood. |
|  | |  |  |  |
| Ridderstrale  2010 | | Military recruits, Sweden | BP: MAP^‡‡‡‡^ at age 20 years positively associated with LVMI at age 40 years.  BMI: Baseline BMI positively associated with follow-up LVMI in a univariable model.  Glycaemic control: Baseline insulin levels positively related to follow-up LVMI in those with elevated baseline BP in multivariable model  Cardiac function analysed in cross-sectional manner. | Longitudinal changes in RFs analysed using two-way ANOVA. RFs related to later LVMI by linear and regression. |
|  | |  |  |  |
| Gardin  2002 | | CARDIA | BP: In a biracial population 28 to 40 years of age, initial SBP significantly positively associated with year 10 LVM in white men and black women and change in SBP positively associated with future LVM in black women.  BMI: Baseline BMI and change in BMI significant positive predictors of 10 year future LVM. | Bivariable Pearson correlation coefficients computed for change in LVM and each RF. Multiple linear regression used to estimate effect of each RF. |
| Lorber  2003 | | CARDIA | BP: In a biracial population 28 to 40 years of age, black men and black women with increasing SBP over 10 years had higher LVMI at 10 year follow-up. For black women the same was true for LVG.  BMI: Black men, black women and white women with increasing BMI over 10 years had higher LVMI.  Glycaemic control: Analysed in cross-sectional manner. | 10 year trends in BMI and BP assessed with year 10 LVM and LVG by fitting for SBP and BMI a separate regression line to the 10 year follow-up for each person and calculating Pearson correlation coefficients between the person-specific slopes estimated from regression models and LVMI and LVG. A linear random coefficients model was used to obtain the person-specific slopes. |
|  | |  |  |  |
| Lieb  2009 | | Framingham  Offspring, USA | BP: In individuals with a mean age of 45 years, increase in SBP was associated with increase in LVMI.  BMI: Increasing BMI associated with greater increments in LVM; association stronger in women.  Glycaemic control: Subjects in low RF group experienced lesser increases in LVM with time. | Long term changes: Multilevel models used to model individual changes in LVM with age over a 16 year period.  Short term change: Four year echocardiographic changes between exams 4-5 and exams 5-6. RFs used from earliest of each consecutive pair of exams. Generalised estimating equations used to allow for fact that some participants contribute twice. To estimate the “overall burden” of RFs, high risk and low risk groups were created (based on presence/absence of hypertension, obesity and diabetes). Median SBP and DBP among the groups used in the regression equation. |
| Cheng  2010 | | Framingham  Offspring, USA | BP: In individuals with a mean age of 45 +/- 10 years, higher SBP, PP^§§§§^ and hypertension treatment significantly related to both greater LVIDD and LVWT ^\|\|\|\|\|\|\|\|^. SBP and PP positively associated with FS^####^; DBP negatively associated with FS and LVIDD.  BMI: Higher BMI significantly related to both greater LVIDD and LVWT.  Glycaemic control: Individuals with diabetes mellitus experienced greater age-associated increases in LVWT and lesser decrease in LVIDD.  Individuals in low RF group experienced greater changes in FS with age compared to those in high RF group. | As for Lieb et al. 2009 |
| Lam  2010 | | Framingham Offspring, USA | BP: In individuals with average ages of 52+/-10 years for men and 51+/-10 years for women in short term group and 46+/-10 years for men and 45+/-10 years for women in long term group (age range 25-74 years), lower SBP and higher DBP associated with greater AoRD***** in mutually adjusted short term change models, also adjusted for BMI, age and sex.  In similar models with PP and MAP replacing SBP and DBP, lower PP and higher MAP associated with greater AoRD.  In long term change models including the same variables, higher DBP was associated with greater AoRD and lower SBP with greater AoRD in men.  In similar models with PP and MAP replacing SBP and DBP, lower PP and higher MAP associated with greater AoRD.  BMI: Higher BMI associated with greater AoRD in short and long term change analyses after adjustment for BP, age and sex. | As for Lieb et al. 2009 |
| McManus  2010 | | Framingham  Offspring, USA | BP: In individuals with an average age of 45 years, participants with higher RF burden (higher SBP) had a greater LAD^†††††^ increase after 16 years. DBP was inversely related to LAD size.  BMI: Participants with a higher RF burden (higher BMI) had a greater LAD increase after 16 years.  Glycaemic control: Analysed in cross-sectional manner. | As for Lieb et al. 2009 |
|  | |  |  |  |
| Jokiniitty  2001 | | Tampere, Finland | BP: Baseline 24h PP in 35 to 45 year old men positively associated with LVMI^‡‡^ 10 years hence. PP stronger predictor of future LVMI than SBP or DBP. Increase in PP over the 10 year period correlated with increase in LVMI.  BMI: The best predictive model for increase in future LVMI included increasing BMI. | 5 clusters of baseline BP variables chosen – casual BP, daytime IAMB^‡‡‡‡‡^, nocturnal IAMB, 24h IAMB and a 5^th^ cluster with all preceding BP variables. Stepwise regression analyses performed to identify models predictive of future LVMI. |
|  | |  |  |  |
| Lauer  1991 | | Framingham, USA | BP: Higher 30 year average SBP positively associated with LVMI and LVH at follow-up (average age of 68 +/- 6 years). 30 year average DBP correlated with LVMI but to a lesser extent than SBP.  BMI: Analysed in cross-sectional manner. | Student’s t test and Pearson correlation coefficients used to estimate associations between RFs and LVM. Multivariable linear regression analyses carried out. |
| Vasan  1995 | Framingham, USA | BP: AoRD in individuals aged 20to 89 years positively associated with MAP and DBP and inversely associated with PP and SBP. Incorporating BP measured 8 years previously in the models yielded minimal changes in r^2^.  BMI: Analysed in cross-sectional manner. | Initially univariable regression analyses carried out determining correlation coefficients for the RF variables with AoRD as the dependent variable. Multivariable regression analyses then carried out. |  |
|  |  |  |  |  |
| Arnlov  2001 | Uppsala, Sweden | Glycaemic control: Fasting glucose at age 50 years was negatively correlated with EF^§§§§§^ at 70 years. | RFs measured at age 50 years related to echocardiography measures at 70 years using partial correlation coefficients. |  |
| Sundstrom  2001 | Uppsala, Sweden | BP: High SBP and DBP at age 50 years predicted prevalence of LVH 20 years later.  BMI: High BMI at age 50 years predicted increased prevalence of LVH 20 years later  Glycaemic control: Analysed in cross-sectional manner. | Multiple logistic regression used to adjust for RFs which were treated as dichotomous variables. ANOVA used to calculate differences in means amongst 4 LVG groups. |  |
| Bjorklund  2002 | Uppsala, Sweden | BP: IVSD, LVPWT, LVMI at age 70 years increased in those with sustained hypertension over previous 20 years. EF not associated with BP. | Logarithmic transformation for skewed variables. ANOVA used to calculate differences in means; potential confounders taken into account by ANCOVA^\|\|\|\|\|\|\|\|\|\|^. |  |
| Arnlov  2005 | Uppsala, Sweden | BP: Higher SBP and DBP at age 50 years associated with decreased E/A ratio after adjustment at age 70 years. Higher SBP associated with higher A-wave, but no association with DBP. No association between either SBP or DBP and LAD.  BMI: Higher BMI at age 50 years was associated with decreased E/A ratio and increased A-wave after adjustment. Higher BMI at age 50 years associated with greater LAD at age 70 years.  Glycaemic control: E-wave at age 70 years positively correlated to fasting glucose at age 50 years. A-wave at age 70 years positively correlated to specific insulin and proinsulin levels at age 50 years. LAD at age 70 years positively correlated to specific insulin and proinsulin levels at age 50 years. | As for Arnlov et al. 2001 |  |
|  |  |  |  |  |
| Strand  2006 | Oslo, Norway | BP: Baseline DBP in men aged 42.1 +/- 0.5 years positively predicted LVM 20 years in the future. SBP was not associated with future LVM.  BMI: Analyses involving baseline BMI and subgroup only documented.  Cardiac function related to subgroups only. | Univariable regression analysis followed by multivariable linear regression analysis. |  |
|  |  |  |  |  |
| Zureik  1995 | Paris, France | BP: 2 year increases in SBP and DBP associated with increases in LVMI and LVWT in men aged 51.6 +/- 2.9 years at baseline.  BMI: 2 year BMI changes not associated with change in LV measurements. | Changes in baseline and follow up values assessed using Student’s t tests. Pearson correlation coefficients used in univariable analysis and multiple linear regression in multivariable analysis. |  |
|  |  |  |  |  |
| Lin  2007 | The Longitudinal Study of Aging, China | BP: Baseline PP positively associated with year 4 LVH in a univariable model in individuals with a baseline age of 71.7 +/-3.9 years.  BMI: Baseline BMI positively associated with year 4 LVMI in a univariable model.  Glycaemic control: Baseline fasting glucose positively correlated with year 4 LVMI.  Cardiac function analysed in cross-sectional manner. | Univariable analysis initially carried out followed by stepwise multiple regression. |  |
|  |  |  |  |  |

*Left Ventricular Mass, †Interventricular septal diameter, ‡Left ventricular posterior wall diameter, §Relative wall thickness, ||Left ventricular internal diameter in diastole, #Left ventricular internal diameter in systole, **Body Surface Area, †† Left ventricular mass index, ‡‡ Systolic blood pressure, §§Left ventricular hypertrophy, ||||Blood pressure, ##Left ventricular posterior wall thickness in diastole, *** Left ventricular posterior wall thickness in systole, †††Analysis of variance, ‡‡‡ Body mass index, §§§Area Under the Curve, ||||||Left ventricle, ###Risk factor, ****Diastolic blood pressure, ††††Left ventricular geometry, ‡‡‡‡ Mean arterial pressure, §§§§Pulse pressure, ||||||||Left ventricular wall thickness, ####Fractional shortening, *****Aortic root diameter, †††††Left atrial diameter, ‡‡‡‡‡ Intra-arterial ambulatory blood pressure, §§§§§Ejection Fraction, ||||||||||Analysis of covariance
